# The changing landscape of discipline in Egypt: a descriptive and predictive study across two generations

**DOI:** 10.1186/s40359-026-05008-0

**Published:** 2026-06-24

**Authors:** Nour M. Zaki, Maya A. Shehata, Jana A. Nasr

**Affiliations:** Department of Psychology, American University in Cairo, AUC Avenue, P.O. Box 74, New Cairo, 11835 Egypt

**Keywords:** Harsh discipline, Parenting practices, Cultural norms, Intergenerational trends, Egypt

## Abstract

**Background:**

Parent–child discipline is often embedded within cultural and social narratives that shape how it is perceived and practiced in different cultures. This study therefore takes an emic approach to understanding discipline in Egypt, with a particular focus on harsh discipline. Specifically, it (1) examines intergenerational continuity and change across discipline domains, (2) explores child, family, and cultural correlates of current discipline practices, and (3) identifies predictors of harsh discipline practices. The sample consisted of 490 Egyptian parents, each of whom had a child between the ages of 2 and 12.5 years.

**Methods:**

Emically-developed scales assessing physical, verbal, psychological, deprivation-based, and positive discipline were administered via an online questionnaire. Harsh discipline was operationalized as the composite of the physical, verbal, and psychological discipline domains.

**Results:**

Findings supported the intergenerational continuity in discipline practices, and identified generational shifts across domains, including lower levels of harsh disciplinary strategies in the second generation. Regression analyses further showed that perceived cultural norms of discipline, recollection of harsh discipline from one’s parents, number of children in a household, and family income significantly predicted harsh discipline.

**Conclusions:**

The findings of this study therefore highlight culturally embedded risk factors for harsh discipline in Egypt. Implications for social, legal, and cultural considerations are further discussed.

**Supplementary Information:**

The online version contains supplementary material available at 10.1186/s40359-026-05008-0.

## Background

While often experienced as a deeply personal interaction between parent and child, discipline is also a fundamentally collective act, one that is heavily influenced by the cultural, social, and political landscapes surrounding the family. The contextual nature of child discipline is evident even in how the term “discipline” is defined and conceptualized. It originates from the word *discipulus*, the Latin word for “pupil.” However, the earliest known use of the word was around the thirteenth century, referring to chastisement of a religious nature, such as self-flagellation [1]. Contemporary definitions of the term also highlight disparate meanings, with some highlighting the notions of punishment or penalties for the sake of obedience [1], and others conceptualizing it as a form of training to cultivate a specific character trait or pattern of behavior [1].

These different conceptualizations of discipline also have histories that are embedded within specific linguistic and cultural traditions. Because the present study adopts an emic approach, the historical framing of discipline in Egypt is more usefully anchored in the semantic field of *taʾdīb* and *adab*. Across Arabic and Islamic intellectual traditions, *adab* has carried meanings related to cultivated conduct, refinement, moral education, and forms of literary-cultural knowledge, while *ta’dīb* has referred to processes of disciplining and ethical-social formation [2, 3]. This semantic history is important because it links discipline to respectability, self-conduct, and social order—themes that continue to shape childrearing expectations in Egypt.

More recently, digitally mediated parenting discourses have increased the visibility of approaches often grouped under labels such as gentle parenting, positive discipline, and respectful parenting. The Covid-19 period appears to have accelerated their circulation through online advice networks, although the broader philosophical roots of positive-discipline approaches predate the pandemic and are commonly traced to the work of Alfred Adler and Rudolf Dreikurs [4, 5]. For the purposes of this study, child discipline will be explored within an Egyptian context, where the literature on disciplinary practices remains notably limited.

### Egyptian culture

Egyptian culture is generally characterized as collectivistic, often prioritizing the group’s needs and social harmony over individual preferences, which can impact child discipline in numerous ways [6]. In Arabic, the term “discipline” translates to the term *تأديب (taʾdīb).* A colloquial variation of this term, *قليل الأدب* (*qalīl al-adab*), is commonly used to refer to someone who is not disciplined or is disrespectful. Interestingly, the exact meaning of the latter term is “someone with little discipline”; however, it is most commonly used to denote deviation from the cultural expectations of respect. For instance, it may refer to an individual who is disrespectful towards elders or someone who uses vulgar language. This linguistic framing is important to highlight as it reflects the association between discipline in Egyptian communities and maintaining the values of the collectivistic culture in which the child is brought up in.

The socially interdependent nature of Egyptian culture also shapes disciplinary practices through its influence on the family structure and caregiving arrangements. For instance, child-rearing is frequently described as a communal endeavor, often involving multiple generations of the family [7, 8]. In many cases, grandmothers are heavily involved in raising children, sometimes in ways that differ from those of the parents’ own approaches, which may reflect their influential role within the family dynamics [9]. Even after marriage, daughters often retain enduring ties to their parents, with many residing nearby, enabling ongoing instrumental care in exchange for their parents’ material support [9]. This extended family support is often necessary as, despite a nationwide decline in fertility rate, many couples still prefer large families as a form of social security [10].

Gender norms further intersect with disciplinary practices in Egypt. Although women have recently been more involved in the workforce, the traditional norm of women’s domesticity in Egypt is rooted in established gender norms. Mothers, wives, and daughters are typically expected to assume primary responsibility for domestic labor and childcare, while men are typically the financial providers [7–9]. The masculine identity, in turn, is often constructed around ideals of resilience, leadership, and emotional restraint [8, 11]. Prominent cultural ideas expect men to fight rather than show weakness or cry [11]. These norms are often reflected on fathers’ parenting, where the paternal role traditionally does not include tending to the child’s emotional needs. Instead, it often focuses on toughening the child and driving them to explore and take risks.

Beyond social and cultural influences, legal frameworks also play a critical role in shaping disciplinary practices in Egypt. While corporal punishment is prohibited in schools, it is still not explicitly prohibited at home [12–14]. Article 7bis of the Child Law allows parents the “right to discipline,” employing ambiguous language that allows certain disciplinary acts provided they are “not deliberate”, thereby limiting the effectiveness of legal protection for children [13, 15]. The lack of legal action against individuals who use corporal punishment as a form of discipline further reinforces cultural views of such practices. Many parents still perceive physical punishment as necessary and legitimate to correct behavior and raise respectful children [14, 16].

Contemporary parenting in Egypt also unfolds within broader conditions of economic strain. Recent macroeconomic pressures, including inflation, rising living costs, and pressure on household consumption, have increased the vulnerability of many families and placed additional strain on children and caregivers [17, 18]. Within family life, such conditions may intensify caregiver stress, reduce emotional and material resources, and increase reliance on immediate coercive strategies in moments of strain. These broader pressures are therefore important to consider when examining how discipline practices are shaped within the Egyptian context.

The OECD’s 2024 Egypt survey documents repeated fiscal packages introduced since 2022 to protect vulnerable groups from rising costs of living and describes inflation and cost pressures on household consumption; UNICEF Egypt’s 2024 annual report also notes that Egypt faced significant macroeconomic and socioeconomic challenges affecting women and children. Taken together, these social, cultural, and legal factors contribute to the normalization of certain discipline practices in Egypt and eventually shape the methods perceived as acceptable and/or effective.

### Discipline trends

Existing literature on discipline in Egypt has highlighted that Egyptian parents tend to use a variety of different methods of discipline, often simultaneously rather than exclusively [19]. Nevertheless, violent forms of discipline appear to be consistently reported across studies. For instance, Youssef et al. found that more than one third of school-age children within their sample had experienced physical discipline (e.g., beatings, burns, or being tied) [19]. More recently, data from the Egyptian Demographic Health Survey indicated that approximately 93% of children had experienced some violent disciplinary practice (e.g., physical punishment and psychological aggression) [20]. Notably, the same survey also documented widespread use of non-violent disciplinary practices. 84.8% of parents had reported explaining to their children that their behavior was wrong, 45.5% reported taking away the child’s privileges, and 37.8% reported redirecting the child to an alternative activity [20]. These findings are important to consider as they highlight that Egyptian parents frequently combine violent and non-violent disciplinary practices, rather than viewing them as mutually exclusive.

This pattern may be understood in light of the broader cultural context in which violence is not always perceived negatively. For instance, attitudes towards practices like capital punishment are shaped by longstanding cultural and legal discourses [21]. In some Egyptian communities, practices like honor killings or female genital mutilation have persisted, often justified through beliefs about safeguarding family honor despite being increasingly opposed by ongoing campaigns and reform initiatives in Egypt [20, 22, 23]. This overarching view of the cultural narratives related to violence may explain why harsh disciplinary practices may be perceived as necessary or beneficial, and that the lack thereof can lead the child to become lazy and spoiled [14]. Given that this study adopts an emic approach to understanding discipline in Egypt, harsh discipline is conceptualized here as the coercive discipline composite formed by the physical, verbal, and psychological discipline domains of the emically-developed scales. This operationalization reflects the clustering of physical punishment, verbal hostility, and psychological aggression within broader violent-discipline frameworks, including those used in national survey work in Egypt [20]. Because physical punishment has been the most commonly examined form of harsh discipline in prior Egyptian and cross-cultural research, a number of the studies reviewed below focus specifically on corporal punishment.

This conceptualization differs from several global perspectives on harsh discipline. For instance, in the United States, some state-level frameworks distinguish physical discipline from physical abuse primarily based on whether the act results in physical injury, alongside contextual considerations such as severity and intent [24]. Other studies highlight that frequency of physical punishment plays a significant role in distinguishing between discipline and abuse [25]. In contrast, the anti-corporal punishment view posits that any use of physical force, regardless of severity, should be considered harmful and abusive to children [26]. As such, there appears to be no consensus in the literature regarding the boundaries of discipline and abuse, further reiterating the need for more culturally-representative research on the matter. Nevertheless, there appears to be greater agreement around the notion that specific characteristics can increase a child’s vulnerability to experiencing harsh discipline or maltreatment.

### Familial, social, and contextual influences on child discipline

These factors may be understood within process-oriented and ecological frameworks of parenting. Prior work has organized influences on parenting into three broad domains: parental factors, child factors, and broader socio-contextual conditions [27]. This framework helps explain why parents’ recollections of discipline, child characteristics, family structure, household income, and perceived cultural norms may each play a role in shaping current disciplinary practices in Egypt. An ecological perspective further situates discipline within the broader family, community, legal, and cultural systems in which parenting occurs.

Among the factors consistently found to influence prevalence rates of corporal punishment is the parent’s perceived norms of discipline. A recent systematic review indicated that parents were found to be influenced by the norms supporting corporal punishment, leading them to use violence in the home [28]. This pattern can partially be attributed to the fact that social pressure to conform to prevailing social norms may often lead to their internalization and, in turn, increase one’s risk for abusive disciplinary practices. The effect of this internalization is explained by Abdel-Fatah who found, using an Egyptian sample, that a parent’s beliefs in the necessity of physical violence increases the likelihood of its use [29].

The same study also found that household economic status influenced parents’ use of severe physical violence (SPV), where increased use of SPV was found among families with lower wealth indices [29]. Similar results have been found in other cultural contexts. In a study conducted in the United States, both direct and mediated relations were observed between socio-economic status and discipline responses [30]. Parents in low-income households tended to more strongly endorse harsh discipline practices and reported higher stress levels, which were in turn associated with more negative perceptions of the child [30]. These findings can indicate that socioeconomic disadvantage may amplify the impact of social norms surrounding disciplinary practices through elevated stress levels and scarcity of resources.

Beyond socioeconomic status, differences in disciplinary practices within rural and urban environments have also been reported. For instance, in Ethiopia, 64% of school children in rural areas reported experiencing bruises or swelling resulting from parental punishment; compared to 21% of children in urban areas [31]. Similar trends have been documented in Egypt, where children whose fathers were originally from rural areas experienced higher levels of corporal punishment [12]. That said, studies in the United States had more mixed findings. A scoping review comparing child maltreatment in rural and urban areas in the US reported no consistent pattern; instead, this prevalence seemed to differ based on race [32]. Collectively, these findings highlight that macro-level cultural factors can often interact with different demographic characteristics to influence disciplinary practices, emphasizing the importance of multi-level frameworks in understanding patterns of harsh discipline.

### The impact of parental demographics

In addition to their cultural context, several characteristics related to the parent may often influence their choice of discipline practices. Firstly, parents frequently draw on the practices they were exposed to during their own childhood. This intergenerational transmission of discipline practices is supported by a number of studies. In a recent review, Xu et al. reported that 16 out of 18 studies published between 2000 and 2023 supported the intergenerational transmission of corporal punishment [33]. Within the Egyptian context, Abdel-Fatah found that mothers who experienced domestic violence either by the husband, parents, or siblings were more likely to use severe physical violence with their children [29]. These findings were also replicated in a study conducted in China, where high parenting stress was found to intensify the intergenerational transmission of corporal punishment and psychological aggression [34]. It is worth noting that the study also reported that the aforementioned moderating effects were stronger for mothers than for fathers, suggesting gendered pathways in the transmission of disciplinary practices [34].

These gender differences are important to note, as they influence several aspects of parental discipline. For instance, Hallers-Haalboom et al. suggest that despite fathers traditionally being the primary disciplinarian, mothers tended to discipline their children more often [35]. Similarly, Straus and Stewart reported that 64% of mothers in their sample used corporal punishment, in comparison to 58% of fathers [36]. A difference was also evident based on the parent’s age, where adolescent parents tended to utilize more spanking than older parents [37]. This finding was partially supported by Straus and Stewart, who found that although there was a general tendency for younger parents to utilize more corporal punishment, the difference was not significant [36]. These parent-related characteristics warrant examination within collectivistic cultures, where risk factors (e.g., stress, younger parental age) may be offset by extended family involvement and community support.

### Influence of child-related characteristics

In addition to the parents’ demographic variables, child-related characteristics also play a critical role in shaping disciplinary practices. It is important to note that “two children in the same family [are] as different from one another as are pairs of children selected randomly from the population” ( [38], p. 563). In other words, even among the same household, it is often insufficient to examine the parent’s context without accounting for the child-related factors as well.

For instance, the child’s gender is consistently associated with variation in parental discipline. In a US sample, girls were significantly less likely to experience corporal punishment than boys [39]. Similar findings were reported in an Egyptian study, where boys were not only more likely to be subjected to corporal punishment than girls but were also more likely to receive punishment that leaves visible marks [12]. Interestingly, the same study found gender-specific patterns in the behaviors eliciting punishment. Boys were more likely to be punished for hitting their siblings; whereas girls were more likely to be punished for disobedience [12]. These findings underscore the role of discipline in socializing children into the community they are raised in, further stressing the need for culturally-representative research.

Disciplinary practices have been shown to vary depending on the child’s age and developmental stage [35]. The impact of the child’s age is consistently cited in the literature; however, there is a lack of consensus regarding whether harsher discipline is more prevalent among younger or older children. In an Egyptian sample, Abdel-Fatah reported that the chance of experiencing severe physical violence increased with the child’s age [29]. Meanwhile, Straus and Stewart suggest that the use of corporal punishment begins in infancy, peaks between four and five years of age, and then decreases steadily until age 17 [36]. The latter study has been interpreted as reflecting developmental changes in children’s cognitive abilities, whereby parents tend to rely on immediate behavior-focused strategies with younger children, then increasingly shift toward reasoning-based approaches as children mature [40]. Further research is needed to explore whether Abdel-Fatah’s findings would be replicated in other collectivist cultures, or if different patterns may emerge that are more in line with the global trend [29].

Perinatal context may also shape later caregiving practices. In particular, pregnancy wantedness has been linked in some settings to later maltreatment risk, possibly through heightened caregiver stress, ambivalence toward the parenting role, or strain in household resources [41]. This variable may be especially relevant in Egypt given the legal and social constraints surrounding reproductive decision-making, which may shape how unwanted pregnancies are experienced within the family context [42, 43].

In addition to the child’s age, their birth order and family size may also play a role in the disciplinary measures utilized with each child. For example, AboKresha et al. found that, during the Covid-19 pandemic, having a large number of children was a risk factor for violence against children, although this relationship was not consistent across all analyses in their study [44]. That said, similar results have been documented outside the pandemic context. Straus and Asdigian found that chronicity of corporal punishment increased as the number of children increased [26]. In fact, the latter study also proposes that the decline in fertility in the US is part of the explanation for the decrease in corporal punishment over the years [26]. Together, these findings suggest that larger family size seems to be a consistent risk factor for harsh discipline. Meanwhile, findings on birth order are inconsistent. Some studies tend to report more punishment being directed towards middle children [45]. However, Egyptian national surveys report no significant differences in disciplinary practices based on the child’s birth order [20].

#### The present study

Existing Egyptian research has provided important evidence regarding the prevalence of corporal punishment and selected correlates of harsh discipline [12, 14, 20, 29]. However, this literature remains fragmented. Prior studies have often focused on physical punishment, severe physical violence, or limited sets of predictors, and many have relied on broad survey indicators that do not capture the full range of culturally embedded disciplinary practices [12, 14, 20, 29]. In addition, few studies appear to have examined intergenerational continuity, perceived cultural norms, and child- and family-level factors within the same analytic framework. The present study addresses these gaps by using emically-developed discipline scales to examine discipline practices across multiple domains in Egypt, while also identifying child, family, and cultural predictors of harsh discipline. The present study therefore addresses two overarching research questions:

1. To what extent do parents’ current disciplinary practices reflect intergenerational continuity or change when compared to their recollections of how they were disciplined as children?
2. How do parents’ disciplinary practices with their own children, particularly harsh discipline, vary as a function of child, family, and cultural factors within the Egyptian context?

## Methods

### Participants

The sample consisted of 490 Egyptian parents ranging in age from 23 to 62 years (*M* = 37.86, *SD* = 5.77). Of these, 75.3% were mothers and 24.7% were fathers. Participants were originally from different Egyptian governorates. A total of 61.68% were from Cairo, followed by 13.73% from Alexandria, 11.48% from Giza, and the remaining 13.11% distributed across governorates, including Port Said, Suez, Dakahlia, Beheira, Minya, Assiut, Monufia, New Valley, and others.

Regarding current residence, 81.7% of participants lived in urban governorates (i.e. Cairo, Alexandria, Port Said, Suez, Giza), while 11.5% lived in rural governorates, and 6.8% lived abroad at the time of participation. Monthly household income varied across the sample. 24.4% of participants earned below 10,000 EGP a month, 24.8% earned between 11,000 and 20,000 EGP, 15.4% earned between 21,000 and 40,000 EGP, 9.8% earned between 41,000 and 60,000 EGP, 6% earned between 61,000 and 80,000 EGP, 6.4% between 81,000 and 100,000 EGP, and 13.1% earned over 101,000 EGP each month.

The majority of participants were married (93.7%), some were divorced (5.3%), and very few were widowed (0.8%) or separated (0.2%). Most participants lived with their nuclear family (90.2%), while some lived with their extended family (8.4%), and very few lived alone (1.4%). Among nuclear families, 20.4% had one child, 50.2% had two children, 22% had three children, 6.9% had four children, and 0.4% had more than four children.

Each parent had at least one child between the ages of 2 and 12.5 years (*M* = 7.77, *SD* = 2.93). Almost half of the children were male (51%), and half were female (49%). In terms of their birth order, 19.6% of the children were only children, 33.9% were the youngest of their siblings, 10.4% were the middle children, and 36.1% were the eldest.

Parents also reported on their child’s perinatal history. 90.6% of parents reported having wanted the pregnancy with this child, while 3.5% reported not wanting the pregnancy, and 5.9% reported having “no strong feelings” about it. 73.1% of parents had a planned pregnancy, while 26.9% had an unplanned pregnancy. 88.4% of parents reported that, since birth, their child has not experienced any serious medical concerns, while 11.6% of parents reported that their child has. Similarly, 85.5% of parents reported that their child has not received any psychological or physical diagnoses since birth, while 14.5% of parents reported that their child has.

### Procedures

Following the Egyptian research ethical guidelines, the study’s protocol was approved by the Institutional Review Board (IRB) at the American University in Cairo (CASE 2024–2025-096), and the Central Agency for Public Mobilization and Statistics (CAPMAS). The eligibility criteria entailed being an Egyptian mother or a father of a child between the ages of 2 to 12.5 years old. If the parent had more than one child, they were asked to answer questions based on the child they needed the most support with. Participants were recruited using convenience and snowball sampling methods. Recruitment took place through word of mouth, social media platforms, and university networks. Participants first filled out an online consent form via Qualtrics, and then were directed to the online questionnaire. Respondents could only complete the questionnaire once, and those who completed the full questionnaire received an online voucher for an e-commerce platform as compensation for their time. To ensure data quality, the bot detection tools that are available on Qualtrics were activated, and any responses that resembled a bot pattern were eliminated. Responses were also manually reviewed for unusually fast completion times and straight-lining behavior.

### Measures

#### Personal information form

A demographic questionnaire collected information in two domains: (1) parent-related variables, including age, gender, governorate of origin, current residence, household income, household composition, and number of children; and (2) child-related variables, including age, gender, birth order, presence of medical or psychological diagnoses, and perinatal factors (e.g., whether the pregnancy was wanted and/or planned).

Across the three emic discipline scales used in the present study, harsh discipline refers to the composite of the physical, verbal, and psychological discipline domains.

#### Recollection of Parental Discipline Scale (PDS)

This scale consists of 21 items in total, and it assesses five discipline domains with culturally relevant manifestations. This scale was developed for the purposes of this study to capture culturally specific discipline practices that are often underrepresented or absent in Western-developed instruments. The full English version can be found in Supplementary File 1. Two parallel forms were administered, one for recollections of maternal discipline and the other for recollections of paternal discipline. The physical discipline subscale consists of seven items (e.g., “Threw a ‘shebsheb’ [slipper] at me as a form of punishment”), with Cronbach’s alpha coefficients of 0.86 for both maternal and paternal reports. The verbal discipline subscale consists of three items (e.g., “Yelled at me when I did something wrong”), with α = 0.65 for maternal and α = 0.59 for paternal reports. The psychological discipline subscale consists of four items (e.g., “Told me that God would punish me by taking me to hell”), with α = 0.69 for maternal and α = 0.67 for paternal reports. The deprivation of privileges/penalty tasks subscale consists of four items (e.g., “Took away my allowance or toys as a form of punishment”), with α = 0.67 for maternal and α = 0.74 for paternal reports. For all of the first four subscales, higher scores mean an increase in that respective measure of discipline. In contrast, the final subscale measuring positive discipline is reverse-scored, with lower scores indicating higher positive discipline. This subscale includes three items (e.g., “Communicated their expectations to prevent me from repeating misbehavior”), with α = 0.62 for maternal and α = 0.63 for paternal reports.

The scores of the first three subscales (i.e., physical, verbal, and psychological discipline) were combined to form a *Recollection of Harshness of Discipline Scale* (14 items), which demonstrated high internal consistency (*α* = 0.89 for maternal and *α* = 0.88 for paternal harshness reports).

#### Child Discipline Scale (CDS)

This scale was also specifically developed for the purposes of this study, aiming to provide an emic measure of discipline practices in the Egyptian context (see Supplementary File 2). It mirrors the Recollections in Parental Discipline Scale (PDS) in structure and content, but measures parents’ self-reported use of discipline practices with their own children. It includes the same five discipline domains as the PDS. Cronbach's alpha was 0.87 for the child physical discipline subscale, 0.54 for the verbal discipline subscale, 0.67 for psychological discipline, 0.74 for deprivation of privileges/penalty tasks, and 0.59 for positive discipline. Consistent with the PDS, the first three subscales were summed to create the *Child Harshness of Discipline Scale* (α = 0.88).

#### Cultural Norms of Discipline Scale (CNDS)

The CNDS follows the same structure as the PDS and the CDS and it was developed for the purposes of this study (see Supplementary File 3). However, this scale examines participants’ perceptions of the prevalence of disciplinary practices commonly used within their surrounding Egyptian community. As such, respondents were asked to solely report on how common they believe each practice to be (i.e., their perception of their prevalence), independent of their personal agreement with, or endorsement of, these practices. Accordingly, the CNDS was designed to capture perceived community norms rather than personal attitudes or acceptance of specific disciplinary practices.

Cronbach's alpha was 0.93 for the physical discipline subscale, 0.78 for verbal discipline, 0.76 for psychological discipline, 0.79 for deprivation of privileges/penalty tasks, and 0.61 for positive discipline. Physical, verbal, and psychological discipline scores were combined to create the *Perceived Harshness of Discipline Scale*, which demonstrated an internal consistency of *α* = 0.94.

### Analytic plan

Statistical analysis was conducted via SPSS (version 31). Participants missing more than one complete scale were excluded. Item-level missing data were minimal. Missing data within this study was mainly due to incomplete responses at the end of the survey (i.e., one scale in particular). These cases were excluded from analyses involving that scale. Most remaining data contained almost no missing values. Cases were excluded pairwise to maximize the data for all initial analyses. Listwise deletion was utilized for the hierarchical regression. Descriptive statistics were computed for all study variables. To address the first research question regarding patterns of discipline across generations and their associations with different demographic factors, Pearson and Spearman correlations were computed to examine associations between variables. The reported correlation was based on the more appropriate test according to distributional assumptions. MANOVAs were also used to examine differences in discipline practices based on categorical demographic characteristics. To address the second research question, a hierarchical regression analysis was conducted to explore the predictors of harsh child discipline.

## Results

### Descriptives of discipline methods: cultural norms, recollections of discipline in childhood, and current use of discipline with children

For physical discipline, perceived community norms were highest (*M* = 17.02, *SD* = 6.92). This was followed by the level of physical discipline they experienced from their mothers (*M* = 13.48, *SD* = 5.78), then physical discipline from their fathers (*M* = 11.08, *SD* = 5.15). Parents reported the lowest levels of physical discipline with their own children (*M* = 10.19, *SD* = 4.18).

A similar pattern emerged for both verbal and psychological discipline, where perceived norms again appeared to be highest (verbal: *M* = 8.37, *SD* = 3.06; psychological: *M* = 11.89, *SD* = 3.65). This was followed by the verbal and psychological discipline received from mothers (*M* = 6.92, *SD* = 2.53; *M* = 10.27, *SD* = 3.61), then from fathers (*M* = 5.94, *SD* = 2.33; *M* = 9.05, *SD* = 3.51), and finally the verbal and psychological discipline they reported using with their own children (*M* = 5.76, *SD* = 1.98; *M* = 8.04, *SD* = 3.0).

As for the use of deprivation of privileges as a disciplinary measure, perceived norms again appeared to be the highest (*M* = 12.91, *SD* = 3.45). This was followed by the deprivation they reported using with their own children (*M* = 9.62, *SD* = 3.13), then the deprivation they experienced from their mothers (*M* = 9.16, *SD* = 3.13), and finally the deprivation they experienced from their fathers (*M* = 8.50, *SD* = 3.34).

In contrast, positive discipline showed a reversed pattern. Parents reported the highest use of positive discipline with their own children (*M* = 6.27, *SD* = 2.26), followed by perceived cultural norms (*M* = 7.38, *SD* = 2.32), recollections of maternal positive discipline (*M* = 7.60, *SD* = 2.56), and, finally, recollections of paternal positive discipline (*M* = 8.05, *SD* = 2.79). It is important to note that higher scores on the positive discipline scale indicate lower use of positive discipline, as the scale is reverse-scored.

When examining total harshness of discipline, perceived cultural norms of harshness of discipline were highest within participants’ communities (*M* = 37.28, *SD* = 12.64), followed by maternal harshness of discipline (*M* = 30.68, *SD* = 10.42), and paternal harshness of discipline (*M* = 26.07, *SD* = 9.45). Finally, the discipline they reported using with their own children appeared to be the least harsh of all the scales (*M* = 23.99, *SD* = 7.90) (Table 1).

**Table 1:**
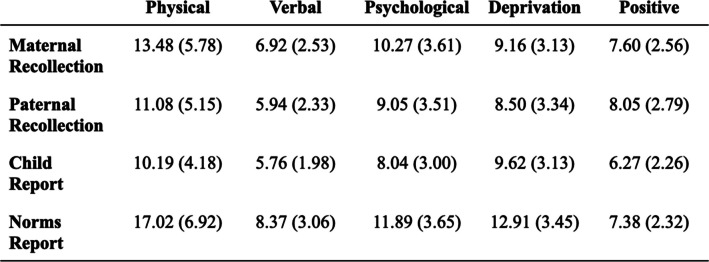
Descriptive results of the four discipline scales

All values refer to mean scores with the standard deviation in parenthesis

### Parents’ recollection of discipline from their own parents

While recollections of maternal physical, verbal, psychological discipline, and deprivation of privileges were generally higher than those of paternal discipline, differences in recollections of maternal discipline emerged based on the respondent’s gender (Pillai's Trace = 0.06, *F*(5, 484) = 6.39, *p* < 0.001, partial η2 = 0.06). In response to the discipline received from their mothers, female respondents reported significantly higher maternal verbal discipline (*F*(1, 488) = 5.76, *p* = 0.02), while male respondents reported significantly higher maternal deprivation (*F*(1, 488) = 5.74, *p* = 0.02). Differences in recollections of paternal discipline also emerged based on the participant’s gender (Pillai's Trace = 0.08, *F*(5, 484) = 8.12, *p* < 0.001, partial η2 = 0.08). Male respondents reported significantly higher rates of paternal physical discipline (*F*(1, 488) = 29.29, *p* < 0.001), psychological discipline (*F*(1, 488) = 13.36, *p* < 0.001), and deprivation (*F*(1, 488) = 19.17, *p* < 0.001). Meanwhile, no significant differences were found in recollections of discipline measures based on the participants’ age (physical discipline: maternal *r* = −0.07, *p* = 0.13; paternal *r* = 0.02, *p* = 0.72; verbal discipline: maternal *r* = −0.07, *p* = 0.14; paternal *r* = −0.08, *p* = 0.10; psychological discipline: maternal *r* = 0.01, *p* = 0.77; paternal *r* = 0.00, *p* = 1.00; deprivation: maternal *r* = −0.06, *p* = 0.16; paternal *r* = −0.05, *p* = 0.25; positive discipline: maternal *r* = 0.01, *p* = 0.76; paternal *r* = 0.01, *p* = 0.81).

Respondents’ household income level was negatively associated with recollections of maternal and paternal discipline. Specifically, maternal physical discipline increased as family income decreased (*r* = −0.15, *p* < 0.001). Similarly, paternal physical discipline, psychological discipline, and deprivation all increased as income level decreased (*r* = −0.19, *p* < 0.001; *r* = −0.19, *p* < 0.001; *r* = −0.15, *p* < 0.001).

Geographic patterns (i.e., governorate of origin and current area of residence) were also identified. With regards to maternal discipline, significant differences between groups emerged based on participants’ governorate of origin (Pillai's Trace = 0.03, *F*(5, 482) = 2.45, *p* = 0.03, partial η2 = 0.03). Participants originally from rural governorates reported significantly higher physical discipline from their mothers than those from urban governorates (*F*(1, 486) = 11.15, *p* < 0.001). Other types of maternal discipline did not differ by one’s governorate of origin (verbal discipline, *p* = 0.08, psychological discipline *p* = 0.15, deprivation *p* = 0.12, positive discipline *p* = 0.18). Significant differences in discipline also emerged based on participants’ area of residence (Pillai's Trace = 0.04, *F*(10, 928) = 1.95, *p* = 0.04, partial η2 = 0.02). Participants currently residing in rural areas reported the highest maternal physical discipline, followed by urban residents, with the lowest levels reported by those currently residing outside Egypt (*F*(2, 467) = 4.13, *p* = 0.02). Other types of maternal discipline did not differ by one’s governorate of origin (verbal discipline *p* = 0.44, psychological discipline *p* = 0.06, deprivation *p* = 0.46, positive discipline *p* = 0.48).

Recollections of paternal discipline also significantly differed based on participants’ governorate of origin (Pillai's Trace = 0.04, *F*(5, 482) = 3.79, *p* = 0.002, partial η2 = 0.04). Individuals from rural governorates reported higher physical (*F*(1, 486) = 18.38, *p* < 0.001), verbal (*F*(1, 486) = 5.68, *p* = 0.02) psychological (*F*(1, 486) = 6.59, *p* = 0.01), and deprivation-based discipline (*F*(1, 486) = 4.59, *p* = 0.03). Recollections of paternal physical and psychological discipline followed the same residential pattern as maternal physical discipline (Pillai's Trace = 0.06, *F*(10, 928) = 3.07, *p* < 0.001, partial η2 = 0.03; physical discipline *F*(2, 467) = 10.89, *p* < 0.001; psychological discipline* F*(2, 467) = 7.27, *p* < 0.001). They were highest among individuals currently residing in rural areas, followed by urban areas, and lowest among those residing abroad. Paternal verbal discipline also differed based on current area of residence (*F*(2, 467) = 3.26, *p* = 0.04). However, it was highest among individuals in rural areas, followed by those living abroad, and lowest in urban areas. A Tukey test further identified a significant difference in paternal verbal discipline between urban and rural residents (*p* = 0.03). However, there was no significant difference between those living in urban areas and abroad (*p* = 0.98), neither between those living abroad and those living in rural areas (*p* = 0.17).

### Parents’ reports of discipline with their own children

Gender differences among participants were identified in self-reported discipline with one’s own child (Pillai's Trace = 0.05, *F*(5, 461) = 4.79, *p* < 0.001, partial η2 = 0.05). Specifically, mothers reported higher use of verbal (*F*(1, 465) = 5.58, *p* = 0.02) and positive discipline (*F*(1, 465) = 6.73, *p* = 0.01) than fathers. When examined by the child’s gender, the MANOVA did not reveal a significant overall effect (Pillai's Trace = 0.01, *F*(5, 461) = 1.18, *p* = 0.32, partial η2 = 0.01). However, between-subject effects indicated that mothers reported using more deprivation with sons than with daughters (*F*(1, 465) = 4.91, *p* = 0.03).

Parental age was positively correlated with certain disciplinary practices. Among mothers, older age was correlated with higher use of psychological discipline (*r* = 0.18, *p* < 0.001), and deprivation (*r* = 0.12, *p* = 0.02). Similarly, among fathers, older age was correlated with higher use of psychological discipline (*r* = 0.22, *p* = 0.02). The child’s age was positively correlated with their parent’s use of physical discipline (*r* = 0.14, *p* = 0.003), verbal discipline (*r* = 0.12, *p* = 0.01), psychological discipline (*r* = 0.15, *p* = 0.001), and deprivation (*r* = 0.18, *p* < 0.001). However, this correlation was not significant for the parents’ use of positive discipline (*r* = −0.04, *p* = 0.43).

Household income level also appeared to have an impact on the discipline measures utilized with one’s own child. Specifically, income was negatively correlated with the use of physical discipline (*r* = −0.14, *p* = 0.002), psychological discipline (*r* = −0.17, *p* < 0.001), and positive discipline (*r* = −0.26, *p* < 0.001). In other words, lower income was associated with higher use of physical and psychological discipline, and lower use of positive discipline (as higher scores on the latter scale are indicative of lower enactment of positive discipline).

Parents’ use of discipline practices with their children also differed based on governorate of origin (Pillai's Trace = 0.05, *F*(5, 459) = 4.47, *p* < 0.001, partial η2 = 0.05). Parents originally from urban governorates utilized significantly more deprivation (*F*(1, 463) = 3.98, *p* < 0.05), as well as positive discipline (*F*(1, 463) = 6.07, *p* = 0.01) with their children. Regarding current residence, the MANOVA did not reveal a significant overall effect (Pillai's Trace = 0.04, *F*(10, 884) = 1.59, *p* = 0.10, partial η2 = 0.02). However, between-subject effects indicated significant differences in deprivation-based discipline; specifically, parents residing in rural areas reported the lowest deprivation, followed by those living abroad, and finally those living in urban areas (*F*(2, 445) = 3.19, *p* = 0.04).

Perceived community norms for discipline were strongly associated with parents’ own practices. Positive correlations were found between each discipline type and its perceived norm: physical discipline (*r* = 0.26, *p* < 0.001), verbal discipline (*r* = 0.40, *p* < 0.001), psychological discipline (*r* = 0.30, *p* < 0.001), deprivation (*r* = 0.30, *p* < 0.001), and positive discipline (*r* = 0.29, *p* < 0.001).

Household composition (i.e., nuclear family, extended family, single-parent) was not significantly associated with discipline practices (Pillai's Trace = 0.01, *F*(10, 922) = 0.629, *p* = 0.79, partial η2 = 0.01). However, the number of children within the household was positively correlated with the parents’ use of physical discipline (*r* = 0.29, *p* < 0.001), verbal discipline (*r* = 0.26, *p* < 0.001), psychological discipline (*r* = 0.31, *p* < 0.001), deprivation (*r* = 0.20, *p* < 0.001), and negatively associated with positive discipline (*r* = 0.11, *p* = 0.02). Furthermore, the discipline methods differed significantly based on the child’s birth order amongst their siblings (Pillai's Trace = 0.12, *F*(15, 1383) = 3.84, *p* < 0.001, partial η2 = 0.04). Physical discipline (*F*(3, 463) = 8.92, *p* < 0.001), verbal discipline (*F*(3, 463) = 10.21, *p* < 0.001), psychological discipline (*F*(3, 463) = 12.29, *p* < 0.001), and deprivation (*F*(3, 463) = 5.31, *p* = 0.001) all appeared to significantly differ based on the child’s birth order, with only children experiencing the lowest scores and middle children the highest across all subscales. Meanwhile, positive discipline did not significantly differ based on the child’s birth order (*F*(3, 463) = 1.50, *p* = 0.21).

A few perinatal and medical demographics were also examined in the context of child discipline. No significant differences in discipline were observed based on whether the pregnancy was planned (Pillai's Trace = 0.01, *F*(5, 461) = 1.14, *p* = 0.34, partial η2 = 0.01). However, participants’ discipline practices with their children also differed based on pregnancy wantedness (Pillai's Trace = 0.04, *F*(10, 922) = 1.98, *p* = 0.03, partial η2 = 0.02). Specifically, physical (*F*(2, 464) = 4.71, *p* = 0.01) and psychological discipline (*F*(2, 464) = 3.39, *p* = 0.04) differed based on pregnancy wantedness, with higher use of these discipline methods when the pregnancy was initially unwanted. Regarding medical diagnoses, the MANOVA did not reveal a significant overall effect (Pillai's Trace = 0.02, *F*(5, 461) = 1.44, *p* = 0.21, partial η2 = 0.02). As for psychological diagnoses, differences between groups emerged (Pillai's Trace = 0.03, *F*(5, 461) = 2.76, *p* = 0.02, partial η2 = 0.03). That said, between-subject results indicated that parents reported greater use of positive discipline with children diagnosed with either a medical (*F*(1, 465) = 6.57, *p* = 0.01) or psychological condition (*F*(1, 465) = 9.50, *p* = 0.002).

Finally, strong associations emerged between parents’ recollections of discipline from their parents, and their choice of discipline with their own children. Recollections of both maternal and paternal physical discipline (maternal *r* = 0.39, *p* < 0.001; paternal *r* = 0.35, *p* < 0.001), verbal discipline (*r* = 0.31, *p* < 0.001; *r* = 0.28, *p* < 0.001), psychological discipline (*r* = 0.36; *p* < 0.001; *r* = 0.39, *p* < 0.001), deprivation (*r* = 0.32, *p* < 0.001; *r* = 0.27, *p* < 0.001), and positive discipline (*r* = 0.29, *p* < 0.001; *r* = 0.26, *p* < 0.001) were all significantly correlated with parents’ use of that method of discipline with their children.

### Hierarchical regression predicting harshness of discipline with one’s children

Based on the composite Child Harshness of Discipline score, bivariate analyses (e.g., Spearman correlations and one-way ANOVAs) were conducted to identify potential predictors of harsh disciplinary practices. Significant associations were found for recollection of maternal harshness (*r* = 0.38*, p* < 0.001), recollection of paternal harshness (*r* = 0.38, *p* < 0.001), perceived cultural norms of harshness (*r* = 0.30, *p* < 0.001), household income (*r* = −0.12, *p* = 0.01), child age (*r* = 0.17, *p* < 0.001), number of children in the household (*r* = 0.34, *p* < 0.001), child birth order (*F*(3, 463) = 12.93, *p* < 0.001), and pregnancy wantedness (*F*(2, 464) = 4.61, *p* = 0.01). These variables were subsequently entered into a hierarchical regression model predicting harsh discipline towards one’s own child. Assumptions of linearity, independence of residuals, normality of residuals, and absence of multicollinearity were met; however, some heteroscedasticity was observed. Therefore, bootstrapping with 1,000 resamples was used for more robust estimates of significance.

The hierarchical regression was conducted in four blocks to evaluate the incremental variance in harshness of discipline explained at each step. In block 1, predetermined child variables were entered (i.e., pregnancy wantedness and birth order). Block 2 added contextual child variables (i.e., child’s age, household income, number of children in the household). Block 3 introduced parental variables (i.e., participants’ recollections of maternal and paternal harsh discipline during their own childhood). Finally, block 4 included parents’ perceptions of cultural norms of harsh discipline. The results of the hierarchical regression are presented in Table 2.

**Table 2:**
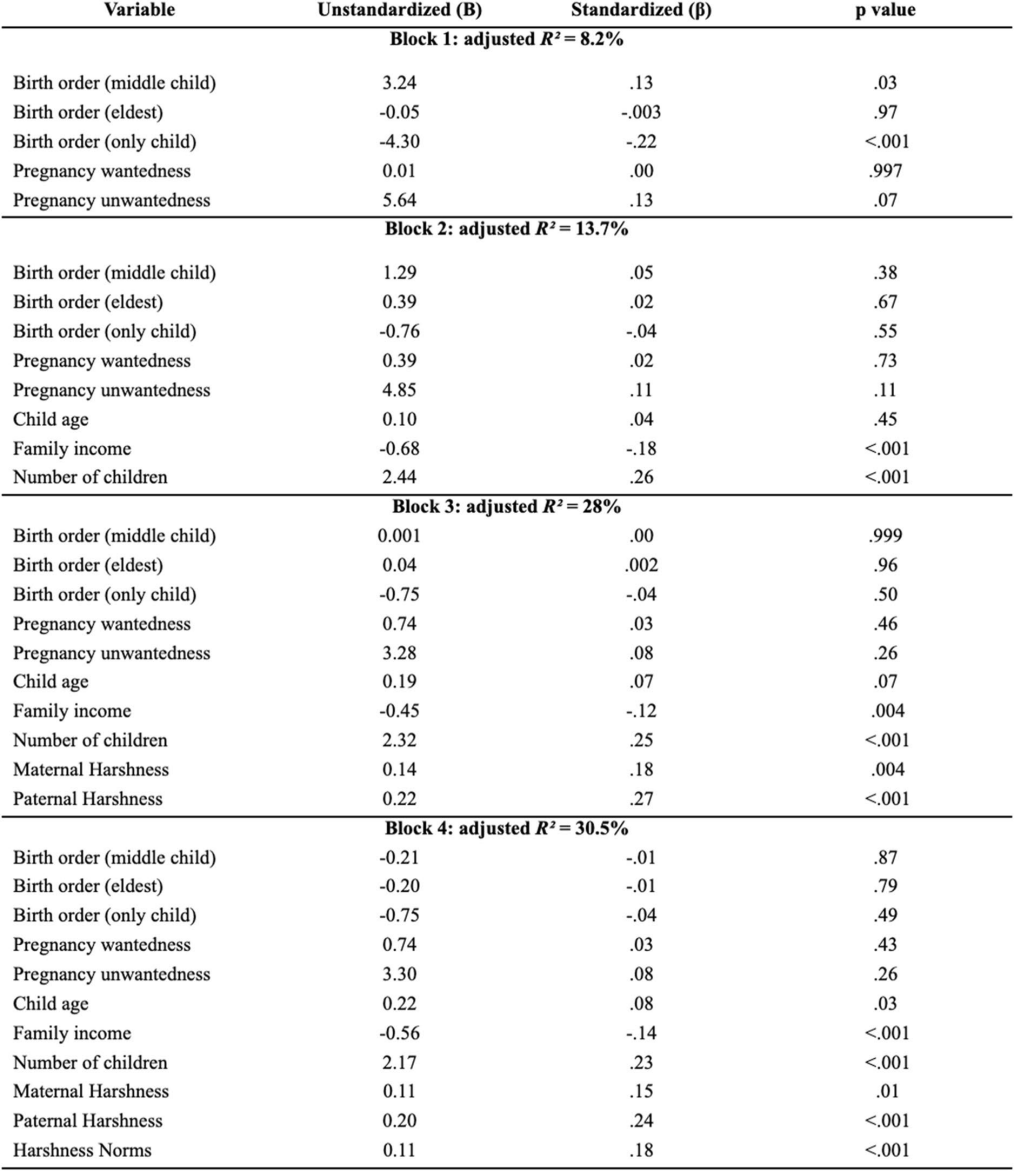
Hierarchical regression analysis predicting harshness of child discipline

#### Block 1: predetermined child variables

Birth order and pregnancy wantedness were dummy coded given their categorical nature. Block 1 explained 9.2% of the variance in harshness of child discipline, *R*^*2*^ = 0.092, Adjusted *R*^*2*^ = 0.082, *F*(5, 451) = 9.18, *p* < 0.001. Regarding birth order, results indicated that being the middle child was a significant positive predictor of experiencing harsh discipline (*β* = 0.13, *p* = 0.03), while being an only child was associated with less harsh discipline (*β* = −0.22, *p* < 0.001). Being the eldest child was not a significant predictor. Similarly, pregnancy wantedness was not a significant predictor of harsh discipline (wanted *p* = 0.997; unwanted *p* = 0.07).

#### Block 2: contextual child variables

Adding contextual child variables (i.e., child age, household income, number of children in the household) in block 2 accounted for an additional 6% of the variance in harsh child discipline. Overall, the model explained 15.2% of the variance, *R*^*2*^ = 0.152, Adjusted *R*^*2*^ = 0.137, *F*(8, 448) = 10.05, *p* < 0.001. Household income was negatively associated with harsh discipline (*β* = −0.18, *p* < 0.001), suggesting that lower income predicted an increased use of harsh discipline. The number of children in the household was positively associated with harsh discipline (*β* = 0.26, *p* < 0.001), indicating that as the number of children in the household increased, so did the parent’s use of harsh child discipline. The child’s age did not significantly predict harsh discipline in this model (*β* = 0.04, *p* = 0.45). Notably, birth order effects were no longer significant once the number of children in the household was included (middle child *p* = 0.38, only child *p* = 0.55), suggesting shared variance.

#### Block 3: parental variables

Adding recollections of maternal and paternal harshness of discipline explained an additional 14.3% of the variance, with the model accounting for 29.5% overall, *R*^*2*^ = 0.295, Adjusted *R*^*2*^ = 0.280, *F*(10, 446) = 18.70, *p* < 0.001. Recollections of both maternal and paternal discipline significantly predicted harsh discipline with one’s child (maternal *β* = 0.18, *p* = 0.004; paternal *β* = 0.27, *p* < 0.001).

#### Block 4: cultural variables

The final block added perceived cultural norms of harshness of discipline, which explained an additional 2.7% of variance, bringing the total variance explained to 32.2%, *R*^*2*^ = 0.322, Adjusted *R*^*2*^ = 0.305, *F*(11, 445) = 19.23, *p* < 0.001. Perceived cultural norms of harshness significantly predicted harsher discipline (*β* = 0.18, *p* < 0.001). In other words, as parents perceived norms of harsh discipline within the culture to be higher, they utilized harsher discipline with their own children. Interestingly, in this model, the child’s age emerged as a significant predictor of harsh discipline (*β* = 0.08; *p* = 0.03). Maternal and paternal recollections of discipline, household income, and the number of children in the household all remained significant predictors of harsh discipline within this model, with the strongest predictors being recollections of paternal harsh discipline (*β* = 0.24) and the number of children in the household (*β* = 0.23).

## Discussion

The present study investigated patterns of discipline in Egypt, specifically focusing on three overarching themes: (a) intergenerational continuity and change in discipline practices, (b) current patterns of child discipline as associated with child, family, and cultural factors, and (c) predictors of harshness of child discipline within the Egyptian context. Overall, the findings revealed noteworthy trends in disciplinary practices across generations, while also highlighting some factors that continue to place some children at higher risk for harsh discipline.

### The changing nature of discipline in Egypt

The results of this study indicated that parents’ recollections of parental (maternal and paternal) discipline predicted their use of harsh discipline with their own children. This aligns with previous research suggesting that discipline is often transmitted intergenerationally [34]. From an attachment perspective, this continuity may be explained through individuals’ early working models of caregivers, formed through the ways they were cared for as children. As Hoffman et al. suggest, “many of us are raised for roughly 18 years by the same parent, who cares for us in the same way over time, [which] also reinforces our earliest picture of caregivers” [46, p. 124]. As individuals step into parenthood, their early caregiving representations can be reactivated (often implicitly), resulting in the replication of familiar parenting and discipline practices.

While this finding supports the intergenerational transmission of discipline practices, the study also identified important generational patterns. First, parents reported higher levels of physical, verbal, and psychological discipline in their own childhoods than they reported using with their own children. This suggests a potential decline in harsh disciplinary practices across the two generations within this study. However, further research is needed to establish this more conclusively, as the present findings are exploratory in nature. In contrast, parents reported using more deprivation of privileges/penalty tasks and positive discipline more frequently with their children than they experienced growing up. These findings align with evidence from other cultural contexts documenting declines in corporal punishment [47]. Straus attributes this decline to four primary factors: (1) demographic changes, including couples having fewer children, later marriage, and higher education levels, (2) expansion of human rights, influencing moral judgments about physical discipline, (3) transition to post-industrial economies where interpersonal skills are increasingly valued (a skill often not supported by corporal punishment), and (4) the legitimization of social science, fostering more awareness about parenting practices [48].

Awareness may be particularly influential in Egypt, where public discourse about child development and the harms of harsh discipline has expanded in recent years, accompanied by recognition of alternative strategies. Notably, this awareness appears no longer restricted to urban contexts. Based on the findings of this study, whereas physical discipline was significantly more commonly reported by individuals originally from rural areas in the first generation, this difference was no longer significant in the second generation. This pattern suggests that access to parenting information may be contributing to a broader measurable impact. As Hoffman et al. emphasize, “We are among the first generations to have access to attachment research that allows us to fine-tune our parenting” ( [46], p. 122]). This access to information facilitates the reconsideration of traditional disciplinary methods.

The role of fathers in child discipline also appears to be potentially evolving across generations in Egypt. Specifically, in the first generation, paternal physical discipline, psychological discipline, and deprivation were all significantly higher in male participants. Meanwhile, in the second generation, no differentiation was observed in fathers’ use of discipline with sons compared to daughters. This shift may reflect broader changes in family roles and the nature of fatherhood. Traditionally, Egyptian fathers assumed the role of provider and authority figure, particularly for sons, helping their sons “toughen up” and adopt their masculine role. However, Dette-Hagenmeyer et al. argue that as female employment increases and women’s dependency on men decreases, a greater emphasis is placed on mutual support and co-parenting [49]. These sociocultural changes may account for the observed differences in paternal discipline across generations within this sample.

Despite the recent shifts in discipline practices, some trends have remained stagnant. Specifically, lower income was consistently linked to higher physical discipline across both generations. This association may stem from increased stress in lower-income households, discipline norms within the community, or limited access to parenting resources. Additionally, some parents may use physical discipline because they believe that it is effective, especially when effectiveness is measured as immediate compliance [50]. In lower-income households, children often later work in blue-collar occupations that prioritize compliance with authority and adherence to safety protocols. As such, parents may not perceive instilling these values in their children through corporal punishment as “harmful” or “unnecessary”. Instead, it may be perceived as effective and functional. These findings highlight the importance of examining discipline practices within a broader socioeconomic and cultural context, and designing interventions that tackle the foundation of discipline trends, not only the conspicuous behaviors.

### Delving into current patterns of child discipline

The results of this study revealed distinct patterns of child discipline among specific demographic groups. Firstly, parents who reported initially not wanting their pregnancy demonstrated higher levels of physical and psychological discipline toward their child compared to both parents who desired the pregnancy and those who reported no strong feelings about it. Interestingly, however, pregnancy planning status was not associated with disciplinary practices. This distinction is noteworthy, as prior research in other cultural contexts has identified relations between unintended pregnancies and child maltreatment [41].

One interpretation may be rooted in religious perspectives on childbearing within Muslim communities. For instance, Sapkota et al. found that participants in a Muslim community in Nepal referred to children as the “gift of Allah”, leading some to avoid family planning due to the belief that reproduction should be left to divine will [51]. While opinions on family planning vary and are rapidly changing in many communities, traditional roots of these beliefs may still impact how families perceive unplanned pregnancies. In other words, an unplanned pregnancy is not necessarily experienced as unwanted in many communities. This cultural nuance may explain why unplanned pregnancies did not significantly impact parents’ use of discipline in the present study.

As for unwanted pregnancies, however, these findings are especially important to consider within the Egyptian context, where abortion remains largely illegal under Articles 260–264 of the Penal Code of 1937, with the exception being when it is necessary to save the life of the pregnant woman [42]. Many parents also chose to avoid abortion for religious reasons [43]. As a result, many families carry an unwanted pregnancy to term, potentially perceiving the child as a financial or emotional burden on the family. Such strain may help explain the higher use of physical and psychological discipline observed among these parents. Additionally, in some cases, such discipline may also reflect parental rejection of their caregiving role or heightened stress due to limited resources.

Family size also emerged as a significant factor associated with disciplinary practices. Results indicated that parents with more children were more likely to use physical discipline, verbal discipline, psychological, and deprivation-based discipline, while engaging in less positive discipline. Similar findings were reported by Straus and Asdigian, who observed that the prevalence and chronicity of corporal punishment increased with family size [26]. Straus suggested that this association may be attributed to reduced parental time and energy to monitor and reason with the child, as well as the increased economic and emotional burden that more children may pose on the parents [48]. Straus also posited that larger families may also lead to reduced time for the marital relationship and personal support networks, which in turn may increase parents’ punitive discipline strategies [48]. Another interpretation of this finding may be related to the place children occupy within family economies. In Egypt, intergenerational support remains an important feature of family life, and kin relations are often shaped by expectations of exchange, obligation, and material transfer across generations [8, 52]. Cunningham et al. further note that parental expectations of later-life companionship and assistance from children are an important consideration in family formation decisions, and that a greater number of surviving children is associated with multiple forms of later-life returns [52]. Within this context, having more children may carry meanings tied to future support and family security, particularly under conditions of economic strain, while also increasing the emotional, financial, and practical demands placed on caregivers in everyday life. These pressures may help explain the greater reliance on harsher disciplinary strategies in larger families.

Birth order findings in this study revealed that physical discipline, verbal discipline, psychological, and deprivation-based discipline were all lowest amongst single children, yet highest among middle children. From an evolutionary perspective, Sulloway argued that when resources are scarce, parents tend to preferentially invest in first-borns as they are the first to reproduce, and their last-borns because they are often the last child they will raise [53]. Meanwhile, middle children lack this preferential treatment and often report comparatively lower levels of parental love in childhood [53]. Similarly, Kidwell found that male middle-children often perceive a lack of parental attention and support, as well as unfair treatment in family rule enforcement [54]. As such, due to the limited time and parental resources, harsher disciplinary measures may be more commonly used with middle children.

Results of this study also indicated that from ages 2 to 12, parental use of physical, verbal, psychological, and deprivation-based discipline increased as children grew older. Interestingly, the literature offers no clear consensus on the impact of age on disciplinary practices. For instance, within a US sample, a study has found that corporal punishment begins with infants, is highest for toddlers, and then begins to decrease afterwards [36]. Meanwhile, a study conducted on an Egyptian sample in Assiut documented that within the 10–12 age range, children experienced harsher discipline as they aged [14]. A potential explanation for this discrepancy may reflect cultural differences in perceptions of the parental role as the child grows up. In individualistic cultures, like the United States, corporal punishment may be avoided with older children to protect their autonomy and individuality. In contrast, in collectivistic cultures like Egypt, harsher discipline may be viewed as sometimes necessary during later childhood to ensure alignment with family values and resistance to negative peer pressure.

This cultural difference also highlights the importance of examining the role of cultural norms in shaping discipline practices. Findings of this study indicated that parents who perceived discipline methods to be normative were more likely to employ them with their own children. Such normalization may lead to a reduced perception of harsh discipline’s harm, leading to its continued use. These findings appear to be ubiquitous across cultures; as Klevens and Whitaker mention, “Social norms regarding physical discipline may be the most prevalent risk factor for child abuse in the United States” ( [55], p. 371]). In Egypt and other collectivistic cultures, where conformity to shared norms is highly valued, such practices may become deeply internalized.

In Egypt, this effect may be amplified by the fact that corporal punishment is not officially illegal in home settings. Together, normative acceptance along with the lack of legal prohibition likely contributes to the perpetuation of harsh discipline. These findings highlight the notion that discipline does not occur in isolation. Instead, it occurs within broader social, cultural, and legal contexts, emphasizing the need for multi-level interventions grounded in the ecological model to comprehensively address the phenomenon.

### Implications: predictors of harshness of discipline and pathways for prevention

Several predictors of harsh discipline identified in this study point to actionable prevention and/or intervention targets across ecological levels. At the macrosystem level, the results indicated that perceived norms of harsh discipline emerged as significant predictors of its use. This draws attention to the need for effective policy and community-level interventions. Policy-makers are urged to establish more explicit prohibitions against the use of corporal punishment towards children at home. Meanwhile, on the community level, advocacy initiatives and public campaigns are needed to shift prevailing attitudes and challenge the norms around harsh discipline practices. It is also important to note that the “cultural spillover theory” posits that violence in one context increases the violence in others [48]. Accordingly, in communities where multiple types of violence are prevalent, it may be important to also advocate for a reduction of all types of violence (e.g., female genital mutilation, domestic violence), as violence against children rarely occurs in isolation.

At the family and community levels, the number of children in the household was also a significant predictor of harsh discipline. From a prevention standpoint, this finding highlights the importance of increased family planning campaigns and outreach, especially in areas where families tend to be larger. From an intervention perspective, families with a larger number of children may benefit from increased social support services to help reduce reliance on harsh discipline measures. Interventions should also prioritize low-income communities, which have demonstrated heightened vulnerability to harsher disciplinary practices. That said, interventions should address contextual stressors (e.g., financial strain, overcrowding of children, parental stress, and limited access to resources), rather than focusing solely on discipline practices.

Finally, at the individual level, parents’ own histories of harsh discipline from their parents emerged as one of the strongest predictors of current harsh parenting. These findings call for an increase in support services (e.g., counseling, parenting programs, support groups) for parents during the preconception, prenatal, and postpartum periods. Such services can facilitate reflection on personal histories of discipline and address concerns parents may have prior to the birth of their child. Examples of these interventions may include the *Towards Parenthood* program, which has been found to reduce parenting stress and depression and anxiety symptoms [56], and the *Mind the Baby* program, which aims to increase parental reflective functioning [57]. Developing new programs or adapting the aforementioned programs in a culturally informed manner may help significantly reduce the rates of harsh discipline utilized after the birth of a child in Egypt. Given that the risk for harsh discipline increases with the child’s age, ongoing developmentally-informed parenting support across childhood may also be critical. These programs would provide parents with access to accurate information about their child’s needs during different developmental stages, while also allowing the parent to reflect on potential triggers associated with that specific stage.

## Limitations & future directions for research

Despite the study’s strengths, several limitations should be acknowledged. While this study examines discipline practices across two generations, its cross-sectional design relies on participants’ recollections of parental discipline and self-reports of their discipline with their own child. This may not be considered a critical limitation as the scales focus on explicit behaviors rather than emotions related to behaviors, making them less prone to bias. However, the bias of self-report remains nonetheless. A longitudinal design following patterns of discipline across generations would provide stronger evidence.

In addition, the total Cronbach’s alpha scores of the harshness of discipline scales were very reliable, some individual discipline subscales demonstrated lower reliability due to having few items. Researchers are recommended to use the total harshness scale rather than the individual subscales in future studies. In addition, it should be noted that measurement invariance across groups was not examined. It is recommended that future studies conduct measurement invariance analyses before making comparisons utilizing this scale. Moreover, while the study included participants from 17 of the 27 Egyptian governorates, the sample primarily consisted of individuals from urban areas. Future studies can aim to utilize sampling methods to enhance representation from rural regions in Egypt.

This study examined birth order as an important variable in predicting harsh discipline. However, previous studies have suggested that shorter spacing between siblings may exacerbate this relationship [54]. This variable was not measured within this study and would be valuable to explore in future research. Finally, given the lack of consensus within the literature on the impact of the child’s age on the harshness of discipline utilized, future studies can examine this relationship through adolescence, as this study caps the age at 12.5 years.

Despite these limitations, this study provides a culturally-grounded examination of discipline in Egypt, offering valuable insight into intergenerational patterns, cultural norms, as well as risk factors. These findings lay the groundwork for culturally-responsive policies and interventions capable of promoting healthier parent–child relationships and fostering lasting intergenerational change.

## Supplementary Information

Supplementary Material 1.

Supplementary Material 2.

Supplementary Material 3.

## Data Availability

The data that support the findings of this study are openly available in Harvard Dataverse Public Repository at [] [https://doi.org/10.7910/DVN/L8TPP0.

## Abbreviations

PDS: Parental Discipline Scale
CDS: Child Discipline Scale
CNDS: Cultural Norms of Discipline Scale
SPV: Severe Physical Violence
IRB: Institutional Review Board
CAPMAS: Central Agency for Public Mobilization and Statistics
