# Supplementary material for: The changing landscape of discipline in Egypt: a descriptive and predictive study across two generations
Source: BMC Psychol. 2026 Jun 24;14:921. doi: 10.1186/s40359-026-05008-0 (PMC13292445; doi:10.1186/s40359-026-05008-0)
Supplement: Supplementary file 1 — Supplementary Material 1. [file 40359_2026_5008_MOESM1_ESM.docx]

**Recollection of Parental Discipline Scale (PDS)**

The following items refer to discipline methods that parents often use with their children. Please respond to each item based on the discipline measures that each of your parents used with you during your childhood.

1 = Never

2 = Rarely

3 = Sometimes

4 = Often

5 = All the time

My mother/father…

1. Threw me with a ‘shebsheb’ as a form of punishment.
2. Grabbed my ear and twisted it as a form of punishment.
3. Stopped talking, responding, or looking at me because they were angry at me.
4. Told me that God would punish me by taking me to hell.
5. Called me a “mistake” or told me that they wish they never had me.
6. Pinched me when I made a mistake.
7. Pulled my hair as a form of punishment.
8. Locked me in my room after I misbehaved.
9. Communicated their expectations to prevent me from repeating misbehavior.
10. Took away my allowance or toys as a form of punishment.
11. Put me in time-out or told me to go to my room when I was acting out.
12. Yelled at me when I did something wrong.
13. Aggressively grabbed or pushed me when I did something wrong.
14. Induced shame in me when I did something wrong.
15. Slapped me when I misbehaved.
16. Used a belt or broomstick to hit me when I did something wrong.
17. Praised me or told me that I did a good job when I behaved according to expectations.
18. Avoided giving me hugs or kisses or saying “I love you” when I did something wrong.
19. Modeled the correct behavior they wanted me to do.
20. Called me names when I made a mistake (e.g., stupid, useless).
21. Punished me by not allowing me to go somewhere I wanted to go.
