# Supplementary material for: The changing landscape of discipline in Egypt: a descriptive and predictive study across two generations
Source: BMC Psychol. 2026 Jun 24;14:921. doi: 10.1186/s40359-026-05008-0 (PMC13292445; doi:10.1186/s40359-026-05008-0)
Supplement: Supplementary file 2 — Supplementary Material 2. [file 40359_2026_5008_MOESM2_ESM.docx]

**Child Discipline Scale (CDS)**

The following items refer to discipline methods that parents often use with their children. Please respond to each item based on the discipline measures that you, as a parent, may utilize to discipline your child.

1 = Never

2 = Rarely

3 = Sometimes

4 = Often

5 = All the time

How often do you…

1. Throw your child with a ‘shebsheb’ as a form of punishment?
2. Grab your child’s ear and twist it as a form of punishment?
3. Stop talking, responding, or looking at your child because you are angry at them?
4. Tell your child that God will punish them by taking them to hell?
5. Call your child a “mistake” or tell them that you wish you never had them?
6. Pinch your child when they make a mistake?
7. Pull your child’s hair as a form of punishment?
8. Lock your child in their room after they misbehave?
9. Communicate your expectations to prevent your child from repeating misbehavior?
10. Take away your child’s allowance or toys as a form of punishment?
11. Put your child in time-out or tell them to go to your room when they are acting out?
12. Yell at your child when they do something wrong?
13. Aggressively grab or push your child when they do something wrong?
14. Induce shame in your child when they do something wrong?
15. Slap your child when they misbehave?
16. Use a belt or broomstick to hit your child when they do something wrong?
17. Praise your child or tell them that they did a good job when they behave according to expectations?
18. Avoid giving your child hugs or kisses or saying “I love you” when they do something wrong?
19. Model the correct behavior you want your child to do?
20. Call your child names when they make a mistake (e.g., stupid, useless)?
21. Punish your child by not allowing them to go somewhere they want to go?
