# Supplementary material for: The changing landscape of discipline in Egypt: a descriptive and predictive study across two generations
Source: BMC Psychol. 2026 Jun 24;14:921. doi: 10.1186/s40359-026-05008-0 (PMC13292445; doi:10.1186/s40359-026-05008-0)
Supplement: Supplementary file 3 — Supplementary Material 3. [file 40359_2026_5008_MOESM3_ESM.docx]

**Cultural Norms of Discipline Scale (CNDS)**

The following items refer to discipline methods that parents often use with their children. Please respond to each item based on the discipline measures that you believe are commonly used within the Egyptian community. Please note that your rating for each discipline measure is not indicative of your level of agreement with the item; it is only a measure of how commonly you think other parents utilize this discipline measure.

1 = Never

2 = Rarely

3 = Sometimes

4 = Often

5 = All the time

1. Throwing the child with a ‘shebsheb’ as a form of punishment?
2. Grabbing the child’s ears and twisting it as a form of punishment?
3. Stop talking, responding, or looking at the child because they are angry at him/her?
4. Telling the child that God will punish them by taking him/her to hell?
5. Calling the child a “mistake” or telling him/her that they wish they never had them?
6. Pinching the child when he/she makes a mistake?
7. Pulling the child’s hair as a form of punishment?
8. Locking the child in a room after he/she misbehaves?
9. Parents communicating their expectations to prevent their children from repeating misbehavior?
10. Parents taking away their children’s allowance or toys as a form of punishment?
11. Putting the child in time-out or telling him/her to go to their room when they are acting out?
12. Yelling at the child when he/she does something wrong?
13. Aggressively grabbing or pushing the child when he/she does something wrong?
14. Inducing shame in their children when they do something wrong?
15. Slapping their children when they misbehave?
16. Using a belt or broomstick to hit their children when they do something wrong?
17. Praising their children or telling them that they did a good job when they behave according to expectations?
18. Avoid giving their children hugs or kisses or saying “I love you” when they do something wrong?
19. Modeling the correct behavior they want their child to do?
20. Calling their children names when they make a mistake (e.g., stupid, useless)?
21. Punishing their children by not allowing them to go somewhere they want to go?
